# Idraparinux or Idrabiotaparinux for Long-Term Venous Thromboembolism Treatment: A Systematic Review and Meta-Analysis of Randomized Controlled Trials

**DOI:** 10.1371/journal.pone.0078972

**Published:** 2013-11-20

**Authors:** Yanzhi Song, Xiaodong Li, Settipalli Pavithra, Dong Li

**Affiliations:** 1 Department of Hematology, BenQ Medical Center, Nanjing Medical University, Nanjing, Jiangsu Province, China; 2 Department of Radiotherapy, BenQ Medical Center, Nanjing Medical University, Nanjing, Jiangsu Province, China; 3 Southeast University, Nanjing, Jiangsu Province, China; Maastricht University Medical Center, The Netherlands

## Abstract

**Background:**

Venous thromboembolism (VTE) is a prevalent disease with potential serious consequences. Idraparinux and idrabiotaparinux are two kinds of long-acting pentasaccharides. Evidence has shown that idraparinux and idrabiotaparinux are effective anticoagulants. However, up to now, there is no consensus on whether they are better than other anticoagulation methods for long-term VTE treatment.

**Objective:**

To evaluate the effect of idraparinux or idrabiotaparinux versus other anticoagulation methods for long-term VTE treatment.

**Methods:**

We searched Cochrane Central Register of Controlled Trials, PubMed, Embase, Web of science, clinical trial registry web sites (clinical trials,WHO clinical trial registry), Googlescholar, PubMed related articles and companies' web sites electronically up to Dec 30^th^, 2012 and manually searched the reference lists and conference proceedings. Only randomized controlled trial (RCT) involving adult patients comparing idraparinux and/or idrabiotaparinux versus other anticoagulation methods for long-term VTE treatment was included. Two reviewers evaluated the studies and extracted data independently. Pooled risk ratios (RRs) were calculated as outcome measures and Revman 5.2 software was used to analyze data. Our primary efficacy and safety outcomes were the recurrent VTE and major bleeding rates.

**Results:**

We included four RCTs and involved 8584 participants on idraparinux or idrabiotaparinux versus standard warfarin for VTE treatment from 9364 references. We did not perform meta-analysis on the VTE rate because of the significant heterogeneity. We used the fixed effect model to analyze the safety outcomes and demonstrated that idraparinux or idrabiotaparinux decreased major bleeding rate significantly (RR 0.73, 95% CI 0.54 to 0.98, P = 0.04) but had a trend to increase the all cause mortality (RR 1.26, 95% CI 1.00 to 1.57, P = 0.05) compared with warfarin.

**Conclusions:**

Until now there is not sufficient evidence to clarify whether idraparinux or idrabiotaparinux is as effective and safe as the standard warfarin treatment for VTE treatment.

## Introduction

Venous thromboembolism (VTE), including the deep venous thrombosis (DVT) and the pulmonary embolism (PE), is a prevalent disease with potentially serious consequences. A major life-threatening complication for VTE patients is the pulmonary embolism that will lead to sudden collapse and death. Even in patients without such an acute condition, in the longer term, they can cause pulmonary hypertension and post-thrombotic syndrome leading to chronic swelling and ulceration of the skin. The incidence of VTE is about seven per 10 000 person-years among community residents [1]. For in hospital patients, it is much more common and the incidence of VTE is more than 100 times greater than the community residents [2]. Now it has become the third most frequent cause of death and the most common preventable cause of hospital death [3], [4].

Even recently the main medicine options in the treatment of venous thromboembolic disorders is the initial course of unfractionated heparin or low-molecular-weight-heparin (LMWH), followed by vitamin K antagonist warfarin for more than three months. However, warfarin has a narrow therapeutic window, requires close monitoring to keep the INR within 2–3 and furthermore has numerous drug interactions that will affect its effect and adverse reactions remarkably.

Idraparinux and idrabiotaparinux are two kinds of long-acting pentasaccharides which are modeled on the pentasaccharide sequence in heparin and activate antithrombin-III after binding with it. They have a long half life [5] and can be injected subcutaneously weekly. Hence, they can improve patients' tolerance and quality of life greatly. On the other hand, the anticoagulative effect may accumulate and the bleeding risk may increase after long term administration [6]. To resolve this problem, idrabiotaparinux was introduced which was a compound of the idraparinux and biotin that could be neutralized immediately by the external avidin. When the avidin is administered, it rapidly binds to the biotin of the idrabiotaparinux. Then the avidin–idrabiotaparinux complex are rapidly cleared from plasma to tissues where avidin normally distributes, resulting in a rapid decrease of circulating anti-FXa activity [7], [8]. Studies [9], [10] have shown that idrabiotaparinux is as effective as idraparinux and can be reversed by avidin at the time of bleeding. Many studies have shown that idraparinux and idrabiotaparinux are effective for long-term VTE treatment, but there is no concensus on whether they are more effective and/or safer than other anticoagulation methods. This systematic review aims to evaluate the therapeutic effects as well as the adverse effects of idraparinux and idrabiotaparinux for long-term VTE treatment.

### Objective

To evaluate the effect of idraparinux or idrabiotaparinux versus other anticoagulation methods for long-term VTE treatment.

## Methods

This meta-analysis followed the PRISMA (Preferred Reporting Items for Systematic Reviews and Meta-Analysis) Statement issued in 2009 (see Checklist S1).

### Inclusion Criteria

Only randomized controlled trials (RCTs) involving adult patients (18 years and over) comparing idraparinux and/or idrabiotaparinux versus other anticoagulation methods (either pharmaceutical or mechanical) for long-term VTE treatment were included. We excluded pregnant patients, hemodialysis patients and patients who had contraindications to anticoagulation treatment. For patients with serum creatinine concentration above 180 mmol/L or glomerular filtration rate less than 30 ml/min who were well hydrated, the anticoagulant doses needed to be adjusted according to the renal function. All participants involved received one of the four treatments: idraparinux (2.5 mg subcutaneously (sc) weekly), idrabiotaparinux (3 mg sc weekly), the standard warfarin treatment and other anticoagulation methods (either pharmaceutical or mechanical).

Our primary efficacy and safety outcome measures were the recurrent VTE and major bleeding rates. Our secondary efficacy outcome measures were the rates of the recurrent DVT, total PE, non-fatal PE and fatal PE. Secondary safety outcome measures were mortality, VTE and bleeding associated mortality and other serious adverse effects rate. DVT was confirmed by either ascending venography, 125-I labeled fibrinogen uptake or Doppler ultrasound. Clinical scoring and D-dimer assay were not acceptable. PE was confirmed by pulmonary angiography, high probability V/Q scan, computerized tomography (CT) or post-mortem. Major bleeding was defined as a bleeding event that resulted in one of the following: retroperitoneal, intracranial, intraspinal or involved any other critical organ, bleeding leading to reoperation or intervention, declined hemoglobin levels more than 2 g per deciliter, a transfusion ≥2 units of packed red blood cells and a bleeding index of 2.0 or more. The bleeding index was derived by summating the number of transfused units of packed red blood cells or whole blood with the difference in hemoglobin levels measured in grams per deciliter before and after a bleeding event as mentioned above.

### Searching Methods

Two reviewers independently searched the Cochrane Central Register of Controlled Trials (CENTRAL) in The Cochrane Library, the PubMed, the Embase, the Web of Science, the clinical trial registry web sites (http://www.clinicaltrial.gov/; http://apps.who.int/trialsearch/. Accessed Dec 30^th^, 2012), the Googlescholar, the PubMed related articles and the companies web sites (GlaxoSmithKline and the Sanofi-Aventis) electronically up to Dec 30^th^, 2012. Searching strategy for PubMed see File S1.

We specified three searching themes.

- To identify VTE related words, we used the terms “vein” “embolism and thrombosis”“hemostasis” “pulmonary veno-occlusive disease”. We also used the free words “vein*”“venous*”“veni*”“vena*”“intraven*”“vessel*”“vascular*”“vasculi*”“pulmonary”“lung” “thrombot*”“thromboe*”“thrombos*”“thrombi*”“clot*”“embol*”“occlusion*”“block*”“hemastas*”“hemostas*”“coagula*”“anticoagula*”“stenos*”“obstruct*”“restenos*”“DVT”“PE”“VTE”.
- To identify idraparinux related words, we used the free words “pentasaccharide”“idraparinux”“idrabiotaparinux”“factor x”“factor 10”“pentasaccharid*”“idraparinux*”“idrabiotaparinux*”“ssr126517”“org34006”“ep217609”.
- To identify RCT studies, we used the Cochrane highly sensitive search filters for identifying randomized trials in Medline and Embase [11].

We also hand searched the related journals, the reference lists of the included and related articles retrieved by electronic searching. We contacted study authors, manufacturers and specialists for further information of unpublished trials. We did not restrict races, languages and regions.

### Data Extraction, Evaluation and Analysis

Two reviewers (YS and XL) extracted data from the included studies (see File S2 and Table 1). They also independently assessed quality of trials using the Cochrane Collaboration recommended tool for assessing risk of bias [12] (see File S2 and Table 2). The tool comprised of seven specific domains (named sequence generation, allocation concealment, blinding of participants and personnel, blinding of outcome assessment, incomplete outcome data, selective outcome reporting and other issues). Studies reported sufficient information to show “low risk” of bias in all of the sequence generation, allocation concealment and blinding of outcome assessment domains were stratified into the low risk of bias group. The rest were stratified into high risk of bias group. Studies with high risks in any other domains were stratified into the high risk of bias group, too. Only data of “low risk” of bias groups were combined. Disagreement was resolved through discussion.

**Table 1:** Characteristics of included studies.

Studies	Multicenter study (yes or no)	Age, mean (SD)	Time	Male, n (%)	Disease	Country	Participant (idraparinux or idrabiotaprinux: warfarin)	Intervention	Outcome	Withdrawl/all randomized (%)
Cassiopea	Yes	Idrabiotaparinux group57·4 (16·5); warfarin group 58·1 (15·8)	Aug 1, 2006 to Jan 31, 2010	Idrabiotaparinux group 829 (52%); warfarin group 823 (51%)	Acute symptomatic PE	38 countries	1599:1603	At least 5 days of enoxaparin 1.0 mg/kg twice per day and then idrabiotaparinux 3.0 mg s.c. weekly for 3–6 months	Recurrence rate of symptomatic VTE	Idrabiotaparinux group 270/1578 (17%); warfarin group 312/1595 (20%)
PERSIST	Yes	Idraparinux 2.5mg* group 58.8 (17.2); warfarin group 60.0 (14·8)	August 1999 to April 2001	Idraparinux2.5mg group 69 (52.7); warfarin group 71 (53.8)	Acute symptomatic and proximal DVT	Germany	131:132	5–7 days enoxaparin 1.0 mg/kg twice per day and then idraparinux (2.5mg, 5mg, 7.5mg, 10mg s.c. weekly) or standard warfarin for 12 weeks*	Recurrence rate of VTE	Idraparinux 2.5mg group 8/131 (6.1%); warfarin group 8/132 (6.0%)
van Gogh-DVT	Yes	Idraparinux group 58.0 (17.3); warfarin group 58.9 (17.0)	May 2003 to November 2004	Idraparinux group 799 (55.0); warfarin group 769 (53.0)	Acute symptomatic DVT	20 countries	1452:1452	Idaparinux 2.5mg s.c. weekly for 3–6 months	Recurrence rate of VTE	Idraparinux group 132/1452 (9.1%); warfarin group 93/1452 (6.4%)
van Gogh-PE	Yes	Idraparinux group 62.2 (16.4); warfarin group 61.6 (16.2)	May 2003 to November 2004	Idraparinux group 525 (47.9); warfarin group 552 (49.3)	Acute symptomatic PE	20 countries	1095:1120	Idaparinux 2.5mg s.c. weekly for 3–6 months	Recurrence rate of VTE	Idraparinux group 126/1095 (11.5%); warfarin group 87/1120 (7.8%)

* There were four doses of idraparinux groups (2.5 mg, 5 mg, 7.5 mg and 10 mg once weekly) and a standard warfarin treatment group in it. We included the 2.5 mg group and the warfarin group as a comparator into our data analysis because it was the routine dose of idraparinux used to treat VTE [14].

SD, standard deviation; VTE, venous thromboembolism; DVT deep venous thrombosis; PE, pulmonary embolism; s.c. subcutaneously.

**Table 2:** Quality assessment of included studies.

studies	Random sequence generation (selection bias)	Allocation concealment (selection bias)	Blinding of participants and personnel (performance bias)	Blinding of outcome assessment (detection bias)	Incom outcome data (attrition bias)	Selective reporting (reporting bias)	Other bias
			All outcomes	All outcomes	All outcomes		
Cassiopea	Low risk	Low risk	Low risk	Low risk	Low risk	Low risk	Unclear risk
PERSIST	Low risk	Low risk	Unclear risk	Low risk	Low risk	Low risk	Unclear risk
van Gogh-DVT	Low risk	Low risk	Unclear risk	Low risk	Low risk	Low risk	Unclear risk
van Gogh-PE	Low risk	Low risk	Unclear risk	Low risk	Low risk	Low risk	Unclear risk

DVT deep venous thrombosis; PE, pulmonary embolism. Supporting Information Legends.

We used the Revman software (Version 5.2. Copenhagen: The Nordic Cochrane Centre, The Cochrane Collaboration, 2012) to analyze data and used the relative risk (RR) as the common measure. We used the Mantel-Haenszel fixed effect model to combine studies without significant heterogeneity (confirmed by the P value of the Chi-squared test >0.10 and I^2^<25%). If the heterogeneity was significant (P≤0.10 and/or I^2^ was ≥50%) we performed the subgroup analysis to explore the heterogeneity. If P≥0.10 and 25%≤I^2^<50%, we decided to choose the fixed effect or random effects models to combine data by discussion. We also used sensitivity analyses to assess the association of the quality of included studies and the clinical characteristics. A two-sided P value less than 0.05 was considered as a significant difference.

## Results

### Search Results

**Figure 1:**
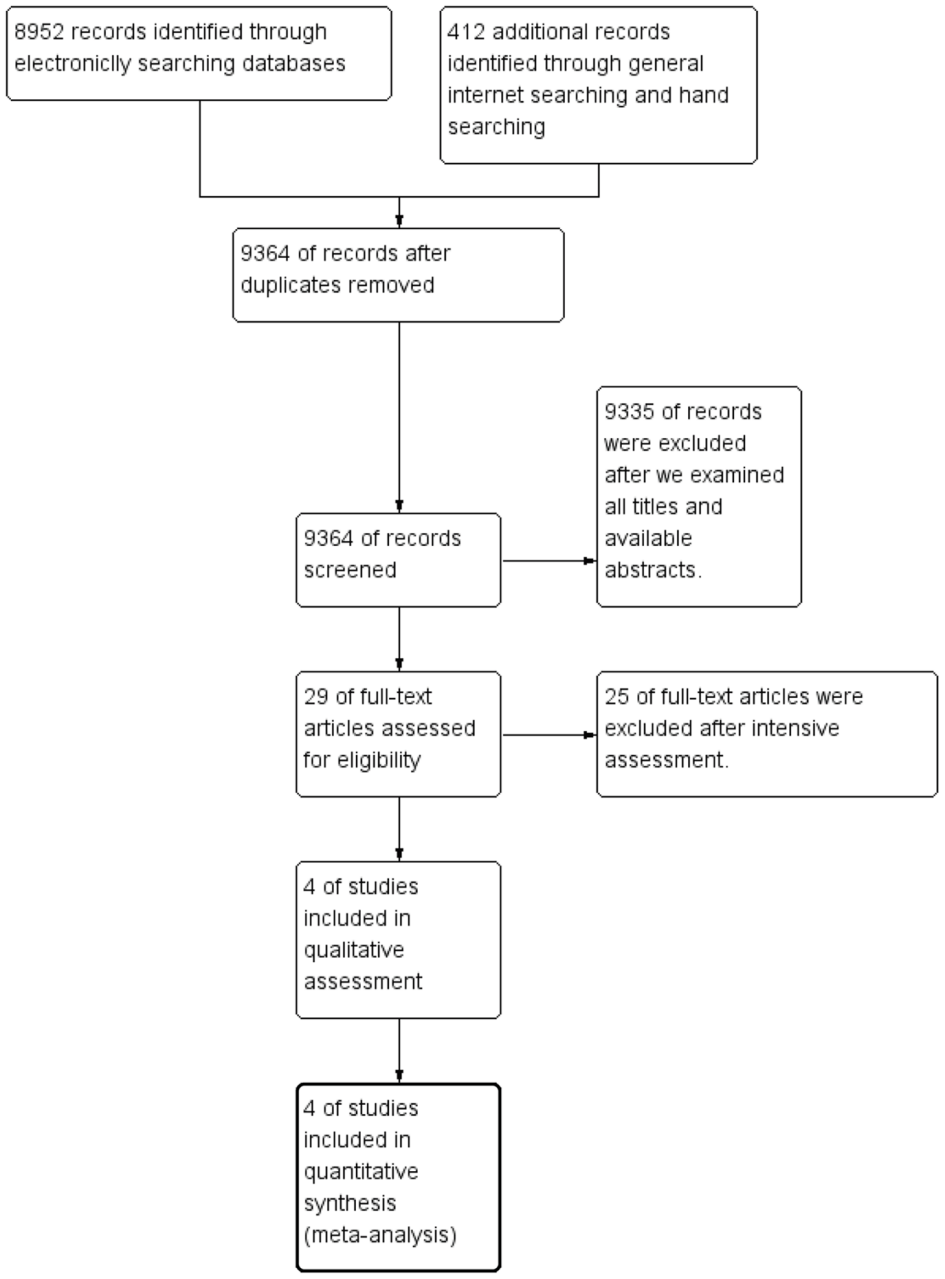
Flow diagram of screening studies for inclusion in systematic review.

The search yielded a total of 9364 references. After we examined all titles and available abstracts, we retained 29 papers. After further assessment, we excluded 25 references which were not RCT studies, studies not relevant to idraparinux or idrabiotaparinux, studies without other anticoagulation methods or placebo as comparators and the duplicate reports. All the reviewers agreed the four studies were included for analysis (diagram see Fig 1).

### Characteristics of Included Trials

All the four included studies (Cassiopea; PERSIST; van Gogh-DVT; van Gogh-PE) [13]–[15] were focused on the idraparinux or idrabiotaparinux versus standard warfarin treatment for long-term VTE treatment. The demographic characteristics of the two treatment groups were similar in the four studies. Two studies (PERSIST and van Gogh-DVT) focused on DVT patients and the rest two (Cassiopea and van Gogh-PE) focused on symptomatic PE patients. The Cassiopea study investigated idrabiotaparinux and the other three studies investigated idraparinux. In the Cassiopea and PERSIST studies, LMWH was injected for a week before idraparinux or idrabiotaparinux was injected, but in the other two studies there was not the LMWH bridge. PERSIST [14] was a phase II clinical trial that involved four doses of idraparinux groups (2.5 mg, 5 mg, 7.5 mg and 10 mg) and a standard warfarin treatment group. We included the 2.5 mg group and the warfarin group as the comparator into our data analysis as 2.5 mg was the routine dose of idraparinux used to treat VTE [14].

### Quality of Included Trials

All the included studies were multi-center RCT studies. Three (Cassiopea; van Gogh-DVT; van Gogh-PE) were multi-country and multi-center studies. All the studies reported the central random sequence generation and reliable blinding of outcome assessment. Van Gogh-DVT, van Gogh-PE and PERSIST were open-label studies, while Cassiopea was a double-blind study. All the included studies were conducted by company. Details of quality assessment of included studies were shown in Table 2 and File S2. As funnel plot should be used with more than 10 studies included we did not use it to detect the publication bias [16], [17]. In the Cassiopea study, the main reasons of withdrawls were adverse events (9% and 11%) and other reasons (6% and 7%) in the idrabiotaparinux and warfarin groups respectively. In the van Gogh- DVT study the reasons of withdrawls were investigator-suspected lack of efficacy (2.2% in the idraparinux group and 1.0% in the warfarin group), adverse events (4.9% and 2.9%) and other reason (2.0% and 2.5%). And in the van Gogh-PE study the reasons were investigator-suspected lack of efficacy (2.0% and 0.3%), adverse events (6.5% and 4.6%) and other reason (3.0% and 2.8%). The withdrawn reasons in the PERSIST study were bleeding complication, suspected recurrent DVT/PE, death, other adverse event, comorbid condition and other reasons, the percentages were not reported.

### Efficacy of Idraparinux or Idrabiotaparinux versus Warfarin

**Figure 2:**
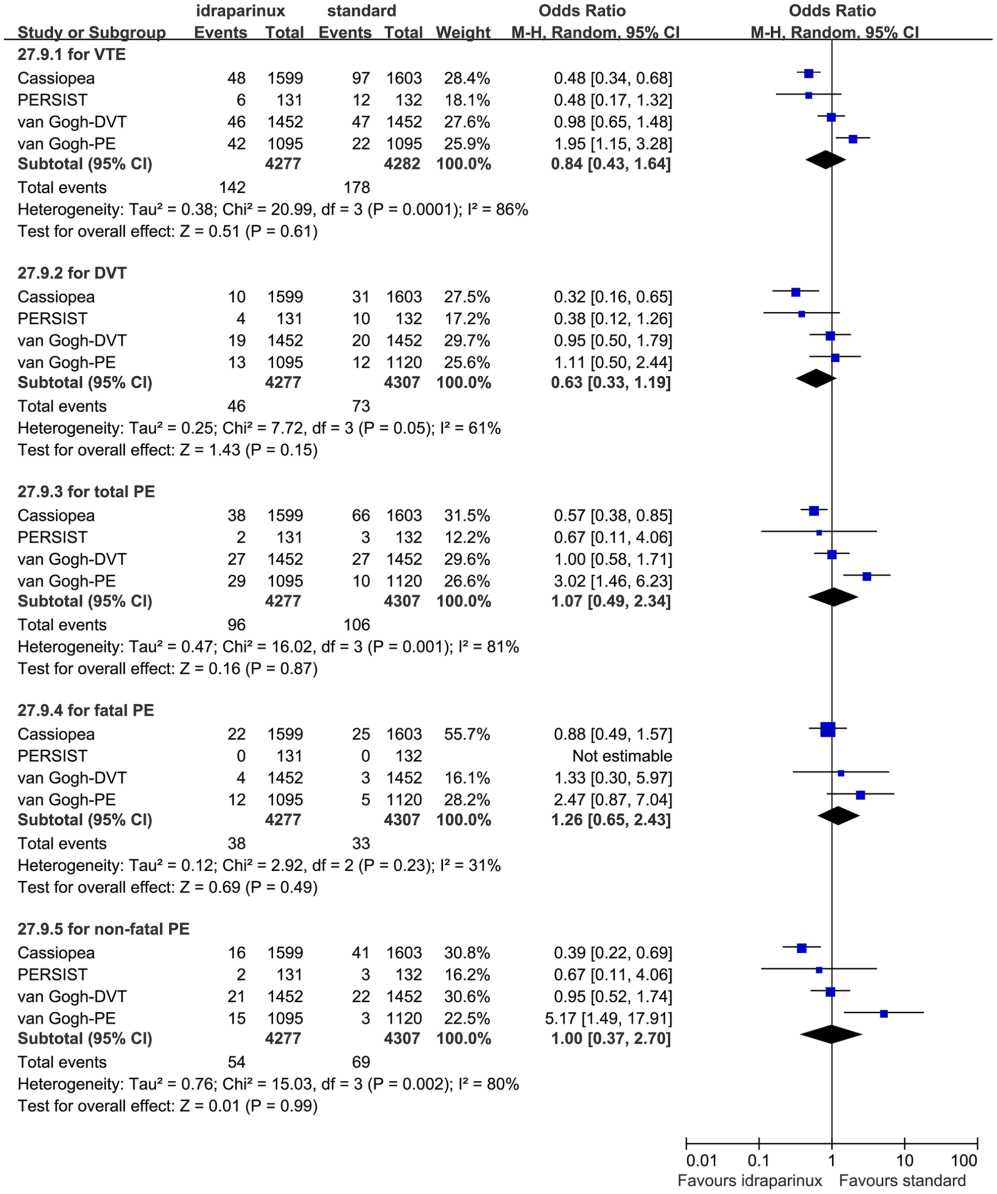
Efficacy of idraparinux or idrabiotaparinux versus standard warfarin treatment.

**Figure 3:**
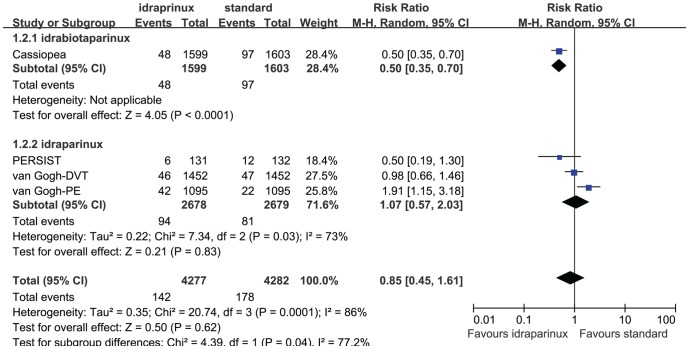
Efficacy medicine subgroup analysis.

The efficacy evaluation result was shown in Fig 2. There was statistically significant heterogeneity in four of the efficacy outcome analyses, the VTE rate (P = 0.0001, I^2^ = 86%), the DVT rate (P = 0.05, I^2^ = 61%), the total PE rate (P = 0.001, I^2^ = 81%), and the non-fatal PE rate (P = 0.002, I^2^ = 80%). Considering there might be clinical heterogeneity we did subgroup analyses to explore it instead of combining all the data into meta-analysis directly. We considered the following subgroups: medicine (idraparinux or idrabiotaparinux), disease (DVT or PE) and followed LMWH or not. The medicine subgroup analysis still showed substantial heterogeneity in the three idraparinux studies (P = 0.03, I^2^ = 73%, see Fig 3). The LMWH subgroup analysis did not show substantial heterogeneity (P = 0.98, I^2^ = 0%) between the two studies combined with the LMWH, whereas substantial heterogeneity between studies without the LMWH bridge (P = 0.04, I^2^ = 76%). The disease subgroup analysis showed relatively non-important heterogeneity (P = 0.21, I^2^ = 37%) between the two DVT studies and substantial heterogeneity between the two PE studies (P<0.0001, I^2^ = 95%,). The heterogeneity of the fatal PE analysis was moderate (P = 0.23, I^2^ = 31%), we combined the data with the random effects model after discussion and calculated the RR (1.26, 95% CI 0.65 to 2.43, P = 0.49, see Fig 2). Because the PERSIST study was a Phase II and was not a large sample study, we excluded it to detect the methodological heterogeneity in the efficacy sensitivity analysis. However, the heterogeneity of the sensitivity analysis was still substantial after we excluded it, yet (P<0.0001, I^2^ = 90%).

### Safety of Idraparinux or Idrabiotaparinux versus Warfarin

**Figure 4:**
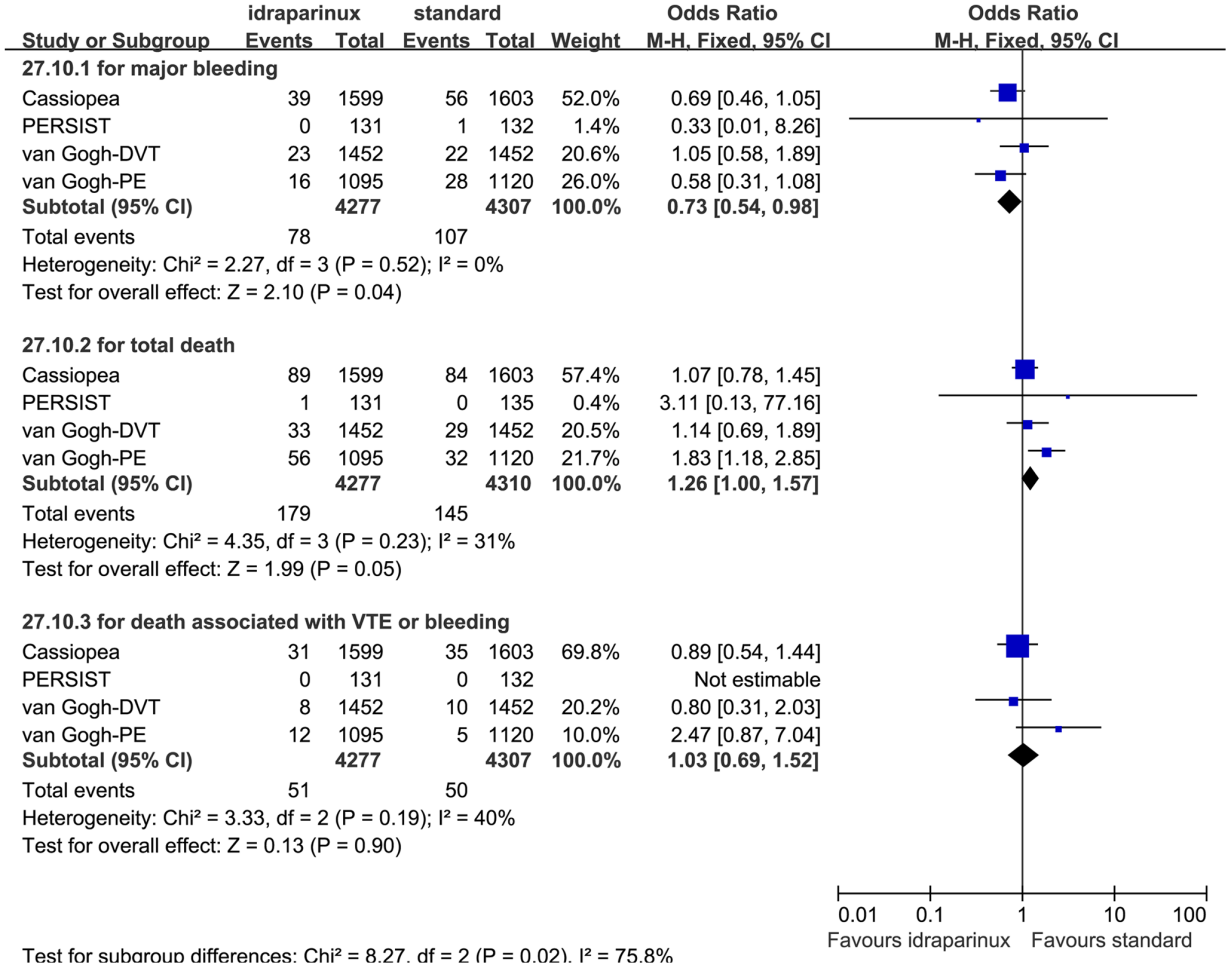
Safety of of idraparinux or idrabiotaparinux versus standard warfarin treatment.

**Figure 5:**
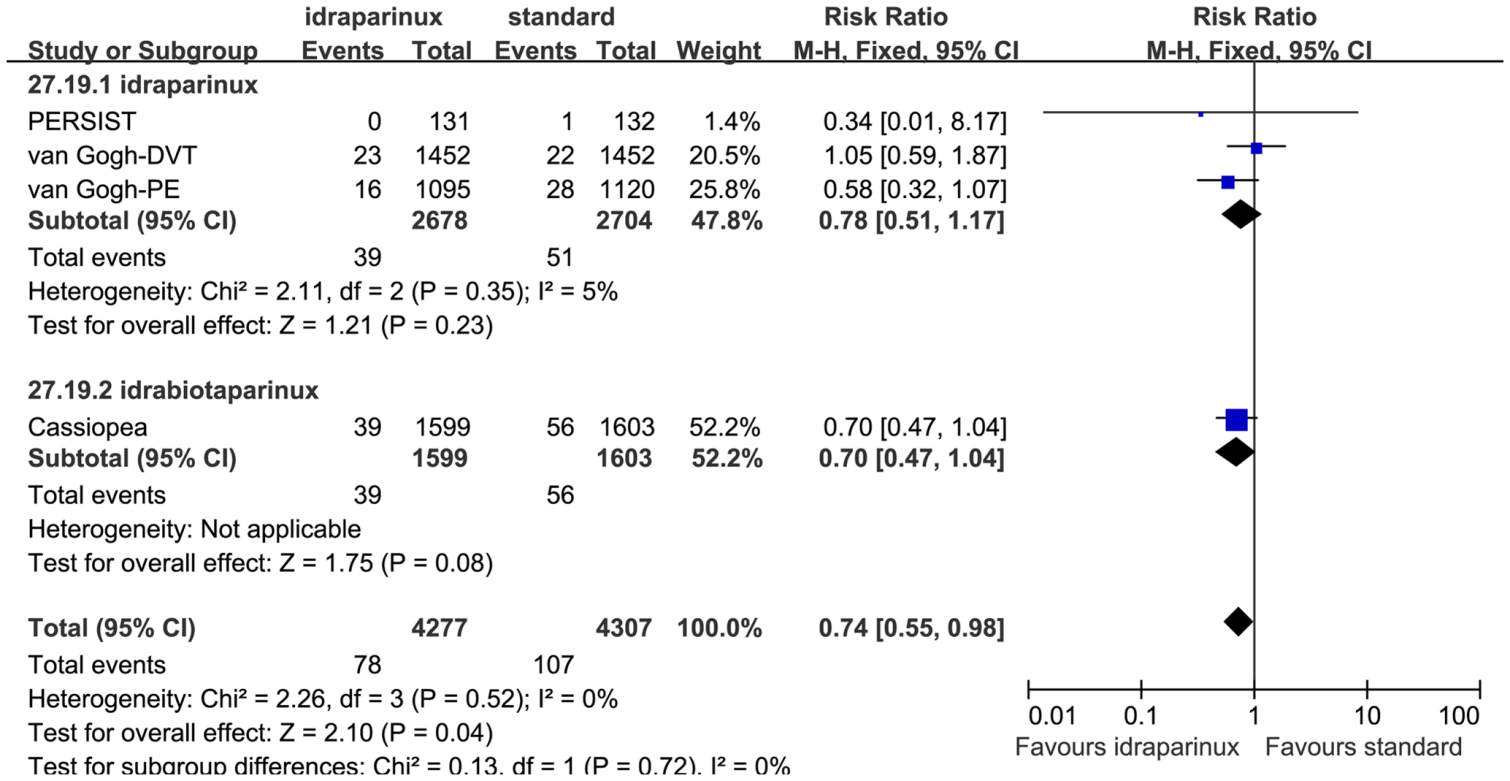
Major bleeding medicine subgroup analysis.

**Figure 6:**
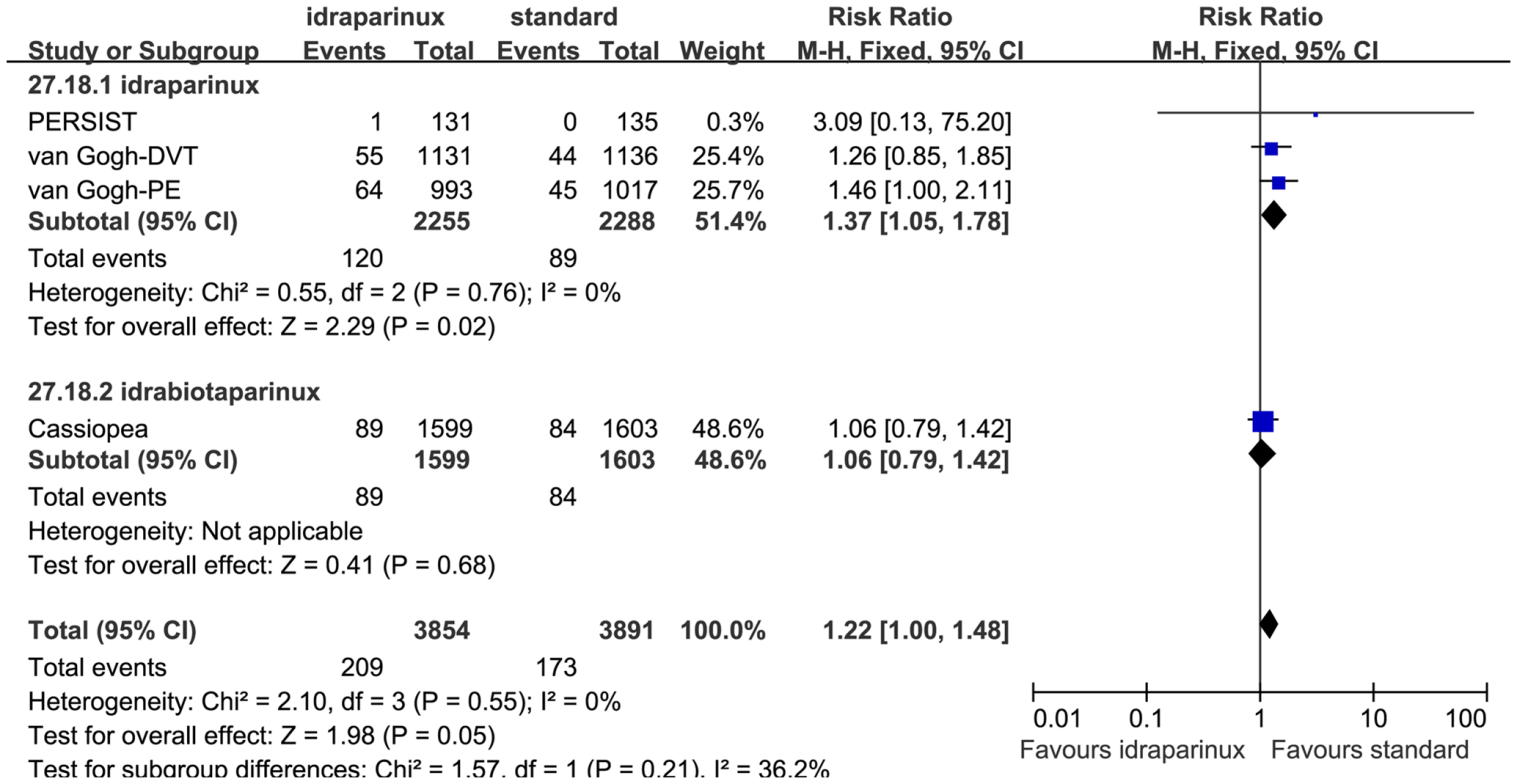
Mortality medicine subgroup analysis.

The safety evaluation result was shown in Fig 4. The heterogeneity of the major bleeding rate analysis was low (P = 0.52, I^2^ = 0%) so we combined them with the Mantel-Haenszel fixed effect model. The heterogeneities of the mortality and the VTE and bleeding associated mortality analyses were moderate, (P = 0.23 I^2^ = 31%) and (P = 0.19 I^2^ = 40%), respectively. After discussion, we analyzed them with the Mantel-Haenszel fixed effect model. Examination of the forest plot revealed that idraparinux or idrabiotaparinux reduced major bleeding rate significantly (RR 0.73, 95% CI 0.54 to 0.98, P = 0.04). But we observed a trend that idraparinux or idrabiotaparinux increased the all cause mortality compared with the standard warfarin treatment (RR 1.26, 95% CI 1.00 to 1.57, P = 0.05). To clarify whether the higher mortality was caused by VTE and bleeding we analyzed the VTE and bleeding associated mortality, but the results were not statistically significantly different between the two arms (RR 1.03, 95% CI 0.69 to 1.52, P = 0.90). As LMWH was not injected as a bridge initially as the usual usage in the van Gogh-DVT and the van Gogh-PE studies, we did a sensitivity analysis that removed the 2 studies. The mortality did not consistently show statistically significant difference between the two arms (RR 1.07, 95% CI 0.80 to 1.43, P = 0.63). There was no other serious adverse effect rate reported in the included studies. The safety sensitivity analyses that excluded the PERSIST study did not show substantial heterogeneity and the results did not change (data not shown). We also did subgroup analyses on the major bleeding rate and the all cause mortality outcomes with the same principle of categorizing subgroups as the efficacy subgroup analyses based on the medicine (see Fig 5,6), the diseases (data not shown) and whether administered after LMWH or not (data not shown). No marked changes were observed on the heterogeneity and the safety outcome measures in these subgroup analyses.

## Discussion

### Efficacy of Idraparinux or Idrabiotaparinux versus Warfarin

Studies in four of the efficacy analyses were substantially heterogeneous regarding to study design and compounds, so we did not do meta-analyses on these outcomes. Only the fatal PE analysis showed unimportant heterogeneity and the result showed similar effect between idraparinux or idrabiotaparinux and the standard warfarin treatment. Nevertheless, the events in this analysis were rare, so the heterogeneity analysis might be powerless to show a statistically significant result. Moreover, the result of the meta-analysis had a so wide confidential interval (0.65, 2.43) that was enough to include a clinically meaningful difference. Therefore, till now, we could not conclude whether the long-acting pentasaccharides are more or less effective compared with the standard warfarin treatment for VTE. In the efficacy sensitivity analysis, after we excluded the only Phase II study the heterogeneity was still substantial. It indicated that the heterogeneity of the studies included in the efficacy analyses was not due to methodological reason [18]. Hence, we considered there might be clinical heterogeneity and performed subgroup analyses to explore it.

When analyzing the medicine subgroups, we found that idrabiotaparinux was more effective than warfarin and idraparinux for VTE treatment. However, only one study was included in the idrabiotaparinux subgroup and it was not sufficient to draw any conclusion. The LMWH subgroup analysis showed that idraparinux or idrabiotaparinux followed LMWH was more effective than the standard warfarin treatment and idraparinux alone. The heterogeneity of the with LMWH subgroup was not important. Besides, we did a meta-analysis that only included the two studies with the LMWH bridge on all the efficacy outcomes and the results also showed statistically significant better effect of the idraparinux or idrabiotaparinux group than the warfarin group (data not shown). The similar result of the related outcomes analyses enhanced the reliability of the result [18]. It implied that idraparinux or idrabiotaparinux was more effective when LMWH was administered first for a week. However, same as the medicine subgroup analysis, the number of studies included in the subgroups was too small to draw firm conclusion. Notably, the inference was based on a comparison between subgroups, but not through directly comparing the two treatment strategies [19]. So this significant association must be further proved in both population-based and hospital-based studies.

### Safety of Idraparinux or Idrabiotaparinux versus Warfarin

The two medicines both have the long half life time, the impaired clearance and/or accumulation of activity of these two compounds may be related to the enhanced bleeding risk [6]. However, our meta-analyses showed that idraparinux or idrabiotaparinux caused less major bleeding compared with the standard warfarin treatment for VTE with non-important heterogeneity. Three of the included studies also demonstrated less major bleeding rate of idraparinux or idrabiotaparinux versus warfarin, although benefit was not statistically significant. The rest study showed that idraparinux and warfarin had similar major bleeding rate, but had a wide confidential interval. In addition, the heterogeneity was not important among the four included trials. The similarity was important and it conferred greater dependability of our result. Therefore, we can draw conclusion that idraparinux or idrabiotaparinux can reduce major bleeding rate than standard warfarin for VTE treatment.

However, to clarify the safety of the two medicines we also should consider the trend of increasing all cause mortality that idraparinux or idrabiotaparinux compared with warfarin. Alternatively, the higher mortality was only reported in the van Gogh-PE study in which participants with symptomatic PE in the idraparinux group received idraparinux initially without LMWH delivered before as usual. Furthermore, the VTE and bleeding associated mortality did not increase and the idraparinux was less effective for PE patients [15], [20]. When we excluded the van Gogh-PE study in a sensitivity analysis the difference of the mortality became non-significant and even the I^2^ value decreased to 0 (data not shown). It suggested that the van Gogh-PE study mainly contributed to the higher mortality. At the same time, in another study included in this analysis, the Cassiopea study which focused on the idrabiotaparinux for symptomatic PE, the mortality of the idrabiotaparinux arm did not increase compared with warfarin arm. Therefore, the conclusion can not be definitively confirmed on whether idraparinux or idrabiotaparinux caused more death versus warfarin according to the evidence available now.

### Comparison with Other Studies

The Amadeus study [21] compared the anticoagulation effect of idraparinux with the standard warfarin treatment to prevent atrial fibrillation patients' thromboembolism events. This study showed that idraparinux was not inferior to warfarin on the efficacy outcome and estimated the hazard ratio 0.71, (95% CI 0.39–1.30, P = 0.007). It was different from our results. In our review we could not conclude whether idraparinux or idrabiotaparinux was as effective as warfarin owing to the substantial heterogeneity in the efficacy analyses. On the other hand, the Amadeus study demonstrated that idraparinux increased major bleeding rate which was inconsistent with our review. We inferred one reason was that the underlying disease in our review was different from the Amadeus study. The other reason was that more participants took antiplatelet medicines contemporarily in the Amadeus study, which increased the bleeding risk. The difference between our review and the Amadeus study suggested that idraparinux or idrabiotaparinux would cause different major bleeding events in varied diseases and clinical settings. And for VTE treatment, it could reduce the major bleeding rate compared with warfarin. However, our results showed a likely higher mortality of the idraparinux or idrabiotaparinux arm than the standard warfarin treatment arm. Similar result was only shown in the van Gogh-PE study [15], but not in the Amadeus study and other studies. Thus the result still need confirming.

We showed better effect of idrabiotaparinux than idraparinux in the subgroup analysis which was a little different from the EQUINOX study [10]. In the EQUINOX study, idrabiotaparinux showed less VTE recurrence and major bleeding rates than idraparinux after the 6 months DVT treatment period, but it was not statistically significant [10]. We considered the difference was caused by the two reasons: first, it was possible that there were not enough participants involved in the EQUINOX study to show the statistical significance. Second, our result was derived from comparison between subgroups, but not by directly comparing the two compounds. To summarise, recent materials are not enough to show which one is better.

### Limitation of the Systematic Review

There were some limitations of our systematic review. First, all of the studies included were conducted by pharmaceutical company and did not use the intention to treat (ITT) analyses, with the inherent conflict of interest and possible bias. Second, we included only four studies so we did not use the funnel plot or other methods to detect the publication bias. Third, three of the included studies were open-labeled that had the risk of compromising concealment allocation [22]. Forth, in three of the four included studies patients were recruited in western countries, only the study Cassiopea involved Asian patients. As Asians were relatively at low risk of VTE [23] and required significantly less heparin to achieve the same goal of anticoagulation compared with other races [24], the efficacy and safety of anticoagulants for Asian patients may be different from the western patients. Finally, there was significant heterogeneity in the efficacy analyses so we did not perform the meta-analyses. Therefore, the reliability of this systematic review might be influenced by these factors, the results had to be interpreted with caution. Although there were limitations, our review was still significant for the future study of the efficacy and safety of the long-acting pentasaccharides for long-term VTE treatment.

Idraparinux and idrabiotaparinux have the long half-life time and can be injected weekly without the need of monitoring coagulation time. They also have rare interactions with other drugs and foods. As a result, they can improve patients' tolerance and adherence and reinforce the feasibility of long-term treatment. Thus they are potential alternatives to warfarin for VTE treatment. Then the data in our review are more correctly reflecting the benefit or harm from the therapy. However, studies on the efficacy analyses were substantially heterogeneous so we could not conclude whether the two medicines were as effective as warfarin. According to our results, idraparinux or idrabiotaparinux could reduce major bleeding rate, but might increase all cause mortality. So we still can not confirm the safety of the two medicines. Our review also demonstrated that the two medicines were more effective if LMWH was injected initially and that idrabiotaprinux was more effective than idraparinux. But the number of included subjects was too small to draw the conclusion. The new oral anticoagulants, the oral direct Factor X inhibitors and the oral thrombin inhibitors, also have shown similar effect as warfarin for long term VTE treatment. As well, they are not in need of monitoring coagulation [25] and are cost-effective [26]. However, problems for these medicines include: hepatotoxicity [27], increase in major bleeding rate and total mortality. There are still not enough data regarding their efficacy for VTE treatment [28]–[31]. Therefore, it is valuable to continue investigating the two long-acting pentasaccharides and more methodological rigorous studies are needed to clarify the efficacy and safety of the two medicines for VTE treatment.

### Conclusions

Until nowadays, There is not enough evidence to conclude whether idrparinux or idrabiotaparinux is as effective as the standard warfarin treatment for long-term VTE treatment. For the safety aspect, they can reduce major bleeding rate but may increase the all cause mortality risk. Therefore, we are still in need of more high quality RCT studies to address the efficacy and safety of the two compounds and to intensively investigate them for patients with VTE diseases in different clinical settings (medicines, diseases, races and with the LMWH bridge or not).

## Supporting Information

Checklist S1 **PRISMA 2009 checklist.**
(DOC)

File S1 **Pubmed searching strategy.**
(DOC)

File S2 **Characteristics of included studies.**
(DOC)
