# Supplementary material for: Idraparinux or Idrabiotaparinux for Long-Term Venous Thromboembolism Treatment: A Systematic Review and Meta-Analysis of Randomized Controlled Trials
Source: PLoS One. 2013 Nov 20;8(11):e78972. doi: 10.1371/journal.pone.0078972 (PMC3835858; doi:10.1371/journal.pone.0078972)
Supplement: File S1 — Pubmed searching strategy. (DOC) [file pone.0078972.s002.doc]

**S2** The Pubmed search strategy

#1 ((((((((((veins) OR vein*[Title/Abstract]) OR venous*[Title/Abstract]) OR veni*[Title/Abstract]) OR vena*[Title/Abstract]) OR intraven*[Title/Abstract]) OR vessel*[Title/Abstract]) OR vascula*[Title/Abstract]) OR vasculi*[Title/Abstract]) OR pulmonary) OR lung 1922752

#2 (((((((((((((((embolism and thrombosis[MeSH Terms]) OR hemostasis) OR thrombot*[Title/Abstract]) OR thromboe*[Title/Abstract]) OR thrombos*[Title/Abstract]) OR thrombi*[Title/Abstract]) OR clot*[Title/Abstract]) OR embol*[Title/Abstract]) OR occlusion*[Title/Abstract]) OR block*[Title/Abstract]) OR hemastas*[Title/Abstract]) OR haemastas*[Title/Abstract]) OR hemostas*[Title/Abstract]) OR haemostas*[Title/Abstract]) OR coagula*[Title/Abstract]) OR anticoagula*[Title/Abstract] or stenos*[Title/Abstract] or obstruct*[Title/Abstract] or restenos*[Title/Abstract] 1354085

#3 ((((#2) AND #1) OR PE) OR DVT) OR VTE 408448

#4 Pulmonary Veno-Occlusive Disease[MeSH Terms] 558

#5 #4 or #3 408619

#6 **(((((((((((((((((org34006) OR ep217609) OR pentasaccharide) OR idraparinux) OR idrabiotaparinux) OR factor x) OR factors x) OR factor 10) OR factors 10) OR factor xa) OR factors xa) OR factor 10a) OR factors 10a) OR pentasaccharid*[Title/Abstract]) OR idraparinux*[Title/Abstract]) OR idrabiotaparinux*[Title/Abstract]) OR ssr126517e) OR ssr126517** 781977

#7 ((((((((randomized controlled trial[Publication Type]) OR controlled clinical trial[Publication Type]) OR randomized[Title/Abstract]) OR placebo[Title/Abstract]) OR drug therapy[MeSH Subheading]) OR randomly[Title/Abstract]) OR groups[Title/Abstract]) OR trial[Title/Abstract]) OR RCT 3090224

#8 ((#7) AND #6) AND #5 10440

#9 animals [mh] NOT humans [mh] 4976619

### #10 (#8) NOT #9 7879
