# Supplementary material for: Idraparinux or Idrabiotaparinux for Long-Term Venous Thromboembolism Treatment: A Systematic Review and Meta-Analysis of Randomized Controlled Trials
Source: PLoS One. 2013 Nov 20;8(11):e78972. doi: 10.1371/journal.pone.0078972 (PMC3835858; doi:10.1371/journal.pone.0078972)
Supplement: File S2 — Characteristics of included studies. (DOC) [file pone.0078972.s003.doc]

**S3.** **Tables of characteristics and risk of bias assessment of included studies**

**Table 1. Characteristics of Cassiopea study**

| Methods | A randomised, double-blind, double-dummy, non-inferiority trial |
| --- | --- |
| Participants | Inclusion: consecutive patientswith confirmed acute symptomatic pulmonary embolism attending 291 centres in 37 countries. Diagnostic eligibility criteria were an intraluminal filling defect in the subsegmental arteries or more proximal pulmonary arteries on spiral CT or conventional pulmonary angiography, a high-probability ventilation-perfusion lung scan, or an inconclusive CT, pulmonary angiogram, or lung scan together with objectively documented deep venous thrombosis. Exclusion: Patients were excluded if they were younger than the legal age to provide informed consent in their country or unable or unwilling to provide informed consent. Patients were ineligible if they had one or more of the following risk factors: active bleeding or high risk of bleeding; calculated creatinine clearance of less than 10 mL/min or end-stage renal failure; malignant hyper tension; known allergy to idrabiotaparinux, idraparinux, avidin, or egg proteins; hypersensitivity to enoxaparin, heparin, pork products, warfarin, or any other contra indication listed in the local labelling of warfarin or  enoxaparin; receipt of therapeutic doses of low-molecular-weight heparin, unfractionated heparin, or fondaparinux for more than 36 h before randomisation; indication for treatment with thrombolysis, embolectomy, or a vena cava filter for the present episode of pulmonary embolism; indication for extended anticoagulant therapy other than pulmonary embolism; life expectancy of less than 6 months; or participation in another  pharmacotherapeutical study within the previous 30 days. Women who were pregnant, breast feeding, or premenopausal and not using effective contraceptive measures were excluded. Also excluded were patients with senility, alcoholism, psychosis, or who were unable to cooperate. |
| Interventions | Idrabiotaparinux group: After at least 5 days of enoxaparin 1.0 mg/kg twice per day, all patients received s.c. injections (3.0 mg idrabiotaparinux or matching placebo) once per week. For patients with a creatinine clearance of less than 30 mL/min, the second and subsequent injections were dosed at 1.8 mg. The treatment endured 3 - 6 months according every patient’s status.  Standard group: patients started s.c. enoxaparin 1.0 mg/kg twice per day as soon as possible after randomisation, warfarin or an identical placebo tablet was started within  24 h and adjusted tomaintain an INR of between 2.0 and 3.0 (target 2.5). The treatment endured 3 - 6 months according every patient’s status |
| Outcomes | **The primary efficacy outcome:** symptomatic recurrent venous thromboembolism, defined as either recurrent pulmonary embolism or deep vein thrombosis within 99 days of randomisation and validated by the adjudication committee. Criteria for the objective diagnosis of recurrent pulmonary embolism were a new intraluminal  filling defect on spiral CT or pulmonary angiography; a cut off of a vessel of more than 2.5 mm in diameter on pulmonary angiography; a new perfusion defect of at least 75% of a segment with corresponding normal ventilation (high probability); inconclusive spiral CT, pulmonary angiography, or lung scan results with objectively documented deep vein thrombosis; or adjudicated fatal pulmonary embolism. The criteria for the objective diagnosis of new deep vein thrombosis were a new noncompressible venous segment or a substantial increase (4 mm) in diameter of the thrombus during full compression in a previously abnormal segment on ultrasonography, or a new intraluminal filling defect on venography.  **The primary safety outcome:** clinically relevant bleeding (classified asmajor or clinically relevant non major) and death from all causes. Bleeding was defined as major if it was clinically overt and associated with a fall in the haemoglobin level of 20 g/L or more, required transfusion of two or more units of red blood cells, or was retroperitoneal, intracranial, occurred in a vital organ or contributed to death. |

**Table 2. Risk assessment of Cassiopea study**

| **Bias** | **Authors’ judgement** | **Support for judgement** |
| --- | --- | --- |
| Random sequence generation (selection bias) | Low risk | Quote: “Randomisation, in blocks of four, was done centrally with a computerized voice response system”  Comment: Probably done. |
| Allocation concealment (selection bias) | Low risk | Quote: “Randomisation, in blocks of four,  was done centrally with a computerised  voice response system”  Comment: Probably done. |
| Blinding of participants and personnel (performance bias)  All outcomes | Low risk | Quote: “Warfarin or an identical placebo  tablet was……or sham ratio”  Comment Probably done. |
| Blinding of outcome assessment (detection bias)  All outcomes | Low risk | Quote: “ by the adjudication committee.”.  Comment: Probably done. |
| Incomplete outcome data (attrition bias)  All outcomes | Low risk | Less than 20% (17% of the idrabiotaparinux group and 20% of the standard treatment group)participants discontinued their treatment prematurely  Comment: Probably done. |
| Selective reporting (reporting bias) | Low risk | The primary efficacy and safety outcomes  listed in themethod section are all reported  Comment: low risk of bias |
| Other bias | Unclear risk | Company sponsored. |

**Table 3. Charateristics of PERSIST study**

| Methods | Randomised controlled trial |
| --- | --- |
| Participants | **Inclusion**: Consecutive patients between 18 and 85 years of age with acute symptomatic proximal DVT confirmed by compression ultrasonography or venography were potentially eligible for the study  **Exclusion**: Exclusion criteria included symptoms for more than 14 days; symptomatic pulmonary embolism; documented DVT within the last 2 years; surgery within the past 10 days; more than 32h of therapeutic anticoagulant treatment; contraindication for  anticoagulants; life expectancy < 3 months; pregnancy; serum creatine > 200 umol/L; and a platelet count <100*109 /L; There was no body weight restriction for eligibility. Participating patients provided written informed consent |
| Interventions | Patients received 1 mg/kg enoxaparin s.c. twice daily for 5 - 7 days and then underwent a baseline assessment of thrombotic burden by compression ultrasonography of both legs and perfusion lung scanning. Thereafter, randomisation to one of four idraparinux  dosages (2.5 mg, 5.0 mg, 7.5 mg and 10 mg s.c. once weekly) or warfarin (INR, 2-3) |
| Outcomes | **The primary efficacy outcome**: VTE recurrence and/or progression confirmed by bilateral  compression ultrasonography, perfusion lung scanning or symptoms  **The primary safety outcome**: major bleeding which included fatal bleeding; bleeding  that was retroperitoneal, intracranial, or intraspinal or that involved any other critical  organ; bleeding leading to reoperation; and overt bleeding with a bleeding index of 2 or  more. The bleeding index was calculated as the number of units of packed red cells or  whole blood transfused plus the haemoglobin values before the bleeding episode minus  the haemoglobin values after the episode (in grams per decilitre) |

**Table 4. R**isk assessment of PERSIST study

| **Bias** | **Authors’ judgement** | **Support for judgement** |
| --- | --- | --- |
| Random sequence generation (selection bias) | Low risk | Quote: “randomisation to one of four SanOrg34006 dosages or warfarin took place, stratified for center and active cancer ……using a central service”  Comment: Probably done. |
| Allocation concealment (selection bias) | Low risk | Quote: “randomisation to one of four SanOrg34006 dosages or warfarin took place, stratified for center and active cancer ……using a central service”  Comment: Probably done. |
| Blinding of participants and personnel (performance bias)  All outcomes | Unclear risk | Quote: “Treatment was……but open for type of drug”  Comment: Probably not done. |
| Blinding of outcome assessment (detection bias)  All outcomes | Low risk | Quote: “The paired baseline and 12-week ultrasonographies and lung scanning results were reviewed by a central adjudication committee, blinded to treatment allocation.”  Comment: Probably done. |
| Incomplete outcome data (attrition bias)  All outcomes | Low risk | Quote: “In 614 (93%) patients the primary efficacy outcome was available for analysis.”  Comment: Probably done. |
| Selective reporting (reporting bias) | Low risk | The primary efficacy and safety outcomes listed in the method section are all reported  Comment: low risk of bias |
| Other bias | Unclear risk | Company sponsored. |

**Table 5. Charateristics of van-Gogh DVT study**

| Methods | Randomised, open-label non-inferiority trial |
| --- | --- |
| Participants | **Inclusion**: Consecutive patients over 18 years of age who presented with acute symptomatic deep venous thrombosis or pulmonary embolism were eligible. Patients presenting with lower-extremity symptoms were considered to be candidates for participation  in the DVT Study. The criteria for deep venous thrombosis were a calf trifurcation or more proximal vein that was not compressible on ultrasonography or an intraluminal filling defect on venography. Patients without chest symptoms in whom deep venous  thrombosis was diagnosed were not routinely tested for pulmonary embolism.  **Exclusion**: Patients were ineligible if they met one or more of the following criteria: receipt of a therapeutic dose of low-molecular-weight heparin or unfractionated heparin  administered formore than 36 hours before randomisation; treatmentwith thrombolysis, embolectomy, or a vena cava filter required for the current episode; another indication for a vitamin K antagonist; pregnancy or breast-feeding; a creatinine clearance of less than 10 ml per minute; uncontrolled hypertension (systolic blood pressure > 180 mmHg or diastolic blood pressure > 110 mm Hg); or a life expectancy of less than 3 months. |
| Interventions | **Idraparinux**: Patients who were assigned to the idraparinux group received a once-weekly s.c. dose of 2.5 mg. For patients with a creatinine clearance of less than 30 ml perminute (as calculated with the Cockcroft-Gault formula), the second and subsequent doses were 1.5 mg  **Standard warfarin treatment**: Patients who were assigned to receive standard therapy received tinzaparin, enoxaparin, or intravenous heparin adjusted for the activated partialthromboplastin  time (ratio, 1.5 to 2.5), followed bywarfarin or acenocoumarol (INR, 2.0 to 3.0), which was started within 24 hours after randomisation. During initial treatment, INRs were determined frequently. Heparin was discontinued when the INR was 2.0 or more for 2 consecutive days and the patient had received at least 5 days of initial treatment. Thereafter, the INR was determined at least once per month |
| Outcomes | **The primary efficacy outcome:** symptomatic recurrent venous thromboembolism, defined as objectively documented recurrent pulmonary embolism, deep venous thrombosis, or death attributed to pulmonary embolism. The criteria for diagnosis of recurrent  pulmonary embolism were one or more of the following findings: a new intraluminal filling defect on spiral CT or pulmonary angiography, a cutoff of a vessel of more than 2.5 mm in diameter on pulmonary angiography, a new perfusion defect of at least 75%  of a segment with corresponding normal ventilation (high probability), a new non-highprobability perfusion defect associated with deep venous thrombosis as documented by ultrasonography or venography, or a new pulmonary embolism confirmed at autopsy.  The criteria for the diagnosis of recurrent deep venous thrombosis were one or more of the following findings: a new non compressible venous segment or a substantial increase (4mm or more) in the diameter of the thrombus during full compression in a previously  abnormal segment on ultrasonography or a new intraluminal filling defect on venography.  **The primary safety outcomes:** clinically relevant bleeding (major or clinically relevant non major haemorrhage) and death from all causes. |

**Table 6. R**isk assessment of van-Gogh DVT study

| **Bias** | **Authors’ judgement** | **Support for judgement** |
| --- | --- | --- |
| Random sequence generation (selection bias) | Low risk | Quote: “with the use of a computerized  voice-response system. Randomization was  stratified according to center and intended  treatment duration”  Comment: Probably done. |
| Allocation concealment (selection bias) | Low risk | Quote: “with the use of a computerized  voice-response system. Randomization was  stratified according to center and intended  treatment duration”  Comment: Probably done. |
| Blinding of participants and personnel (performance bias)  All outcomes | Unclear risk | Open-label study.  Comment: Probably not done. |
| Blinding of outcome assessment (detection bias)  All outcomes | Low risk | Quote: “All suspected outcome eventswere  classified by a central adjudication committee,  whose members were unaware of treatment  assignments.”  Comment: Probably done. |
| Incomplete outcome data (attrition bias)  All outcomes | Low risk | Quote: “was complete in 99.3%of patients  in the DVT Study and 99.1% of patients  in the PE Study.”  Comment: low risk of bias |
| Selective reporting (reporting bias) | Low risk | The primary efficacy and safety outcomes  listed in themethod section are all reported  Comment: low risk of bias |
| Other bias | Unclear risk | Company sponsored. |

Table 7. Characteristics of van-Gogh PE study

| Methods | Randomised, open-label non-inferiority trial |
| --- | --- |
| Participants | **Inclusion**: Consecutive patients over 18 years of age who presented with acute symptomatic  deep venous thrombosis or pulmonary embolism were eligible. Patients presenting  with chest symptoms were considered to be candidates for participation in the PE  Study. Criteria for pulmonary embolism were an intraluminal filling defect in subsegmental or more proximal pulmonary arteries on spiral computed tomography (CT) or pulmonary angiography, a high-probability finding on a ventilation-perfusion lung scan,  or a non diagnostic finding with documented deep venous thrombosis. Patients without chest symptoms in whom deep venous thrombosis was diagnosed were not routinely tested for pulmonary embolism.  **Exclusion**: Patients were ineligible if they met one or more of the following criteria: receipt of a therapeutic dose of low-molecular-weight heparin or unfractionated heparin  administered formore than 36 hours before randomisation; treatmentwith thrombolysis, embolectomy, or a vena cava filter required for the current episode; another indication for a vitamin K antagonist; pregnancy or breast-feeding; a creatinine clearance of less than 10 ml per minute; uncontrolled hypertension (systolic blood pressure >180 mmHg or diastolic blood pressure >110 mm Hg); or a life expectancy of less than 3 months. |
| Interventions | **Idraparinux**: Patientswhowere assigned to the idraparinux group received a once-weekly s.c. dose of 2.5 mg. For patients with a creatinine clearance of less than 30 ml perminute (as calculated with the Cockcroft-Gault formula), the second and subsequent doses were 1.5 mg.  **Standard warfarin treatment**: Patients who were assigned to receive standard therapy  received tinzaparin, enoxaparin, or intravenous heparin adjusted for the activated partial- thromboplastin time (ratio, 1.5 to 2.5), followed bywarfarin or acenocoumarol (INR, 2.0 to 3.0), which was started within 24 hours after randomisation. During initial treatment,  INRs were determined frequently. Heparin was discontinued when the INR was 2.0 or more for 2 consecutive days and the patient had received at least 5 days of initial treatment. Thereafter, the INR was determined at least once per month. |
| Outcomes | **The primary efficacy outcome:** symptomatic recurrent venous thromboembolism, defined as objectively documented recurrent pulmonary embolism, deep venous thrombosis, or death attributed to pulmonary embolism. The criteria for diagnosis of recurrent  pulmonary embolism were one or more of the following findings: a new intraluminal filling defect on spiral CT or pulmonary angiography, a cutoff of a vessel of more than 2.5 mm in diameter on pulmonary angiography, a new perfusion defect of at least 75%  of a segment with corresponding normal ventilation (high probability), a new non-highprobability perfusion defect associated with deep venous thrombosis as documented by ultrasonography or venography, or a new pulmonary embolism confirmed at autopsy.  The criteria for the diagnosis of recurrent deep venous thrombosis were one or more of the following findings: a new non compressible venous segment or a substantial increase (4mm or more) in the diameter of the thrombus during full compression in a previously  abnormal segment on ultrasonography or a new intraluminal filling defect on venography.  **The primary safety outcomes:** clinically relevant bleeding (major or clinically relevant non-major haemorrhage) and death from all causes. |

**Table 8. Risk assessment of van-Gogh PE study**

| **Bias** | **Authors’ judgement** | **Support for judgement** |
| --- | --- | --- |
| Random sequence generation (selection bias) | Low risk | Quote: “with the use of a computerized  voice-response system. Randomization was  stratified according to center and intended  treatment duration”  Comment: Probably done. |
| Allocation concealment (selection bias) | Low risk | Quote: “with the use of a computerized  voice-response system. Randomization was  stratified according to center and intended  treatment duration”  Comment: Probably done. |
| Blinding of participants and personnel (performance bias)  All outcomes | Unclear risk | Open-label study.  Comment: Probably not done. |
| Blinding of outcome assessment (detection bias)  All outcomes | Low risk | Quote: “All suspected outcome eventswere  classified by a central adjudication committee,  whose members were unaware of treatment  assignments.”  Comment: Probably done. |
| Incomplete outcome data (attrition bias)  All outcomes | Low risk | Quote: “was complete in 99.3%of patients  in the DVT Study and 99.1% of patients  in the PE Study.”  Comment: low risk of bias |
| Selective reporting (reporting bias) | Low risk | The primary efficacy and safety outcomes  listed in themethod section are all reported  Comment: low risk of bias. |
| Other bias | Unclear risk | Company sponsored. |
